# Mortality risks associated with short-term exposure to ultrafine particles in London and the West Midlands

**DOI:** 10.1097/EE9.0000000000000449

**Published:** 2025-12-16

**Authors:** Devon Nenon, Gary Fuller, Pierre Masselot, Antonio Gasparrini

**Affiliations:** a Department of Public Health Environment & Society, Environment & Health Modelling (EHM) Lab, London School of Hygiene & Tropical Medicine, London, UK; b Environmental Research Group, School of Public Health, Imperial College London, London, UK; c MRC Centre for Environment and Health, Imperial College London, London, UK

**Keywords:** Air pollution, Mortality, Particulate matter, Ultrafine particles, Time series

## Abstract

**Background:**

Some research has suggested that ultrafine particles (UFP), with an aerodynamic diameter less than 100 nm, might be the most harmful component of ambient particulate matter air pollution on human health. In the United Kingdom, UFP toxicity might also be affected by national vehicle fuel policies passed in 2007. We assessed associations between short-term exposure to UFP and mortality in two metropolitan areas in England.

**Methods:**

We conducted a time-series analysis of 851,726 deaths in London and the West Midlands between 2003 and 2018. UFP was assessed as particle number concentration lagged 0–1 days. We used quasi-Poisson regression to estimate associations between particle number concentration and nonaccidental, cardiovascular, and respiratory mortality in both locations. Secondarily, we conducted an interrupted time-series analysis to evaluate potential impacts of the 2007 fuel policy.

**Results:**

For all mortality outcomes in both locations, risk ratios ranged from 0.991 (95% confidence interval [CI] = 0.965, 1.019) to 1.001 (0.990, 1.011) for a 10,000 particle/cm^3^ increase, and all 95% CIs included the null. Null results persisted when lag periods were extended to 0–5 days, linearity assumptions were relaxed, or when co-pollutants PM_2.5_ and NO_2_ were included. In the interrupted analysis, there was only evidence of a change in risk ratio for respiratory mortality in London (p_difference_ = 0.006), and risk ratios before and after the change point were null.

**Conclusions:**

We found no evidence that suggested that UFP is associated with nonaccidental, cardiovascular, or respiratory death, and little evidence that the fuel policy modifies this association.

**What this study adds**

This study provides the longest known analysis of a short-term association between ultrafine particle air pollution and mortality in the United Kingdom, and the first known study to investigate the possible effect modifying role of the United Kingdom “sulfur-free” diesel fuel policy. While we found no evidence that ultrafine particles are associated with increased mortality and little evidence that the fuel policy modifies this association, the risk of potential exposure misclassification highlights the need for more advanced exposure assessment to evaluate the impacts of these particles.

## Introduction

Air pollution exposure is a leading contemporary health challenge, with the majority of air pollution-related deaths attributable to outdoor particulate matter (PM).^1^ There is a wide body of evidence on the so-called fine (PM_2.5_) or inhalable (PM_10_) components of PM, corresponding to particles with aerodynamic diameters below 2.5 µm and 10 µm, respectively.^2^ However, evidence is much more limited for the smallest particle component, known as ultrafine particles (UFP), despite strong rationale suggesting it as potentially more harmful than the larger particles of the range.^3,4^

UFP have an aerodynamic diameter smaller than 100 nanometers (0.1 µm) and largely come from vehicle combustion.^4,5^ They are much more numerous than larger particles, making up the majority fraction of the particle number concentration, although they are often negligible in terms of mass.^5^ Their small size means they can penetrate more deeply into the body than larger particles, and their relatively higher surface area also provides a greater area for adsorption of reactive chemical compounds, which can elicit oxidative stress and an inflammatory response in the body.^4–6^ Toxicological studies on both animals and humans demonstrate a higher potential for UFP translocation through the body, compared with other PM fractions, and report changes in lung function, allergic response, altered heart rate, vascular thrombogenic effects, and increased inflammation.^4,6,7^

Some epidemiological studies have documented associations in line with this toxicological evidence, including links between UFP and all-cause, cardiovascular, respiratory, and pulmonary mortality, as well as with hospital admissions.^2,6,8^ However, these studies are still relatively few and exhibit inconsistent findings.^2,6,8^ This can be attributed to varying composition and source of UFP particles across time and space, but also to the challenges of making high-quality measurements of UFP, and inconsistencies in study designs, outcome selections, and modeling choices.^6–8^ As a consequence, UFP are nowadays largely unregulated.^2^

In the United Kingdom (UK), two studies have been published on the association between UFP and mortality in London, with the first finding some positive associations between UFP and all-cause, cardiovascular, and respiratory mortality from 2000 to 2005 and the second finding no relationship from 2011 to 2012.^9,10^ It is hypothesized that this apparent contradiction is linked to the introduction of legislation in the mid-2000s affecting UFP concentrations. In 2007, the UK mandated the use of “sulfur-free” diesel fuel, which anteceded a marked decrease in UFP levels in London and the West Midlands.^11^ In addition to concentration, this change is also hypothesized to have altered the composition of the UFP particles, which could in turn change their toxicity.^4,11^

This study aimed to investigate associations between UFP and mortality in London and Birmingham and test the hypothesis of toxicity change related to the 2007 traffic policies. To do so, we conducted an analysis covering a longer period than has been done in previous studies to better characterize the relationship between UFP and mortality in the UK.

## Methods

### Study area

This study is set in the built-up areas of Greater London and the West Midlands, the two largest metropolitan areas in the UK. The areas have populations of nine and three million inhabitants, respectively, representing around an eighth of the UK population.^12,13^ These areas were selected from all UK conurbations based on long-term availability of UFP data.

### Data

We obtained daily time series of UFP from the monitoring network of the UK Department for Environment and Rural Affairs.^14^ Measurement came from one site in London (North Kensington) and two sites in the West Midlands (Birmingham Tyburn and Birmingham Centre), based on their class as urban background monitoring sites and the length of data availability.^14^ Figure S1; https://links.lww.com/EE/A394 shows the years of measurement and data missingness for all urban background sites in the UK. UFP was assessed as particle number concentration (PNC), a proxy of UFP, with a minimum operational sensitivity limit of 7 nm, measured at the end of the study period.^14^ Data were available from 1 January 2003 to 12 November 2018 in London, and from 1 January 2003 to 23 September 2013 in the West Midlands. Measurements in the West Midlands were collected at the Birmingham Centre site before 2009 and Birmingham Tyburn thereafter. Measurements were taken hourly or quarter-hourly, which we aggregated to daily averages subject to a 75% daily completeness criterion to ensure representativeness. We removed all measurements below one, as these were implausible.

We obtained daily counts of nonexternal (nonaccidental), cardiovascular, and respiratory mortality for each area from the UK Office for National Statistics. Selected mortality causes were defined by the International Classification of Disease (ICD) 10 codes, including Chapter A-R, Chapter I, and Chapter J, respectively.^15^

We also gathered daily maximum and minimum temperature measurements from the HadUK-grid database produced by the UK Meteorological Office, which we averaged to a daily mean.^16^ We obtained values of PM_2.5_ and NO_2_ over a 1 × 1 km grid from a spatio-temporal ensemble machine learning (ML) model, described elsewhere.^17^ Area-specific daily series of temperature and pollution data were created using area-weighted averages of the intersecting grid cells. We sourced public holiday dates from a publicly available API, Nager.Date.^18^

### Statistical methods

We estimated the association between UFP and mortality using a quasi-Poisson log-linear time-series model following the standard practice of air-pollution-related mortality studies. UFP was included as a 2-day moving average (lag 0–1) to account for lagged effects, in accordance with previous studies on PM and mortality.^10,19^ Temperature effects were included using distributed lag nonlinear models, allowing lagged effect from 0–3 days as 0 and 1–3 strata, modeling nonlinear effects with natural splines with three knots placed at the 10th, 75th, and 90th percentiles. Seasonal patterns and long-term trends were modeled using a natural spline with 7 degrees of freedom (df) per year, and indicators for day of week and public holidays were also included. We fitted the model separately for each UFP measurement site, then pooled the results from both locations in the West Midlands using fixed-effects meta-analysis.^20,21^

To evaluate the effect of the 2007 traffic policies, we conducted a secondary interrupted time-series analysis.^22^ This was done by adding an interaction between the UFP term and a change point indicator placed at 1 January 2008, following previous evidence on their implementation.^11^

### Sensitivity analyses

We conducted a range of sensitivity analyses to evaluate modeling choices on the main (noninterrupted) model. We first relaxed the linearity assumption in the exposure-response relationship by using a natural cubic spline with a single knot placed at the median PNC value (see Supplementary Materials for details on model selection; https://links.lww.com/EE/A394). We then extended the lag structure to 0–5 days using an unconstrained distributed lag linear model. We also extended the lag period for temperature to 21 days to account for possible longer-term cold effects. Finally, we assessed potential confounding by other pollutants using two-pollutant models, with 0–1 day moving averages of PM_2.5_ and NO_2_ separately added to the model as linear terms.

As an internal validation study, we repeated the full analysis in London within the smaller geographical boundary of the Royal Borough of Kensington and Chelsea, approximately a 12 km^2^ administrative district around the measurement site.

All analyses were conducted in R (version 4.3.1) using the dlnm, openair, mixmeta, and Epi packages.^23–26^ This project was granted a favorable ethical opinion by The London School of Hygiene and Tropical Medicine Ethics Committee (reference 30242).

## Results

UFP data was available for 76% of days in London and 69% in the West Midlands. The final analysis included 851,726 nonaccidental deaths across both areas of study, including 277,710 cardiovascular deaths and 125,562 respiratory deaths. The average UFP concentration was about 12,000 particles/cm^3^ in both conurbations, with a decreasing trend throughout the study period. Table 1 presents descriptive statistics for all variables.

**Table 1.** Median (and IQR) of time series daily data by area

	London (2003–2018)	West Midlands (2003–2013)
Mortality (n)		
Nonaccidental	162 (147, 180)	56 (50, 63)
Cardiovascular	51 (43, 61)	19 (15, 23)
Respiratory	23 (18, 29)	8 (6, 11)
UFP (particles/cm^3^)^a^	12,636 (9,226, 18,412)	12,976 (9,202, 18,474)^b^
Temperature (°C)	11.5 (7.2, 15.9)	10.5 (6.4, 14.7)
NO_2_ (μg/m^3^)	33.0 (25.4, 42.9)	25.1 (18.6, 34.1)
PM_2.5_ (μg/m^3^)	10.8 (8.6, 15.2)	9.3 (7.6, 13.1)

a Missing rates: London: 76%; West Midlands: 69%; By site: Birmingahm Centre (2003-2009): 68%; Birmingham Tyburn (2009–2013): 70%.

b By site: Birmingham Centre (2003–2009): 15,437 (11,070, 22,100); Birmingham Tyburn (2009–2013): 10,562 (7,771, 14,405).

We found no evidence of an association between PNC and nonaccidental, cardiovascular, or respiratory mortality in either location (Table 2 and Table S1; https://links.lww.com/EE/A394). For a 10,000 particle/cm^3^ increase in PNC, risk ratios ranged from 0.991 (0.965, 1.019) for respiratory mortality to 1.001 (0.990, 1.011) for non-accidental mortality, both in the West Midlands.

**Table 2.** Relative risk and 95% confidence interval associated with a 10,000 particle/cm^3^ increase in PNC over 0–1 days with adjustment for seasonality, long-term trend, and temperature

	London	West Midlands^a^
Nonaccidental	0.998 (0.992, 1.005)	1.001 (0.990, 1.011)
Cardiovascular	1.000 (0.989, 1.011)	0.992 (0.975, 1.010)
Respiratory	0.997 (0.980, 1.013)	0.991 (0.965, 1.019)

a Pooled.

**Figure 1.**
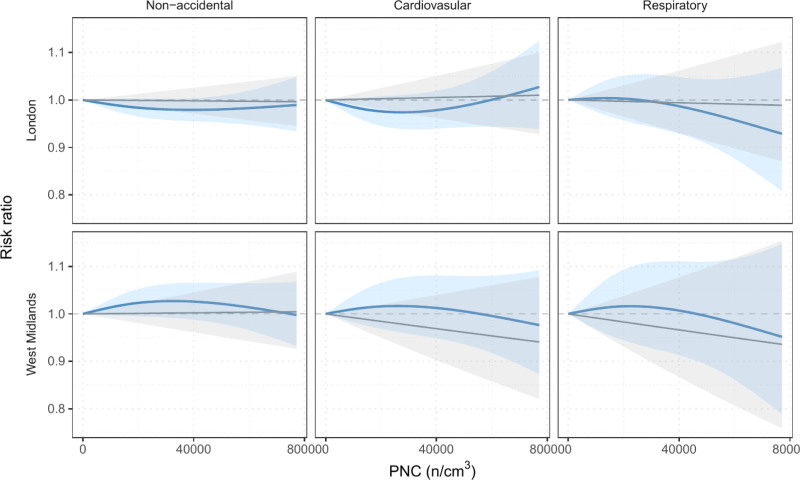
Secondary analysis on the short-term association between UFP and nonaccidental and cause-specific mortality. Exposure-response curves with 95% confidence intervals obtained using a linear term (gray line) or a natural cubic spline (blue line) with a single knot placed at the median (13,160 n/cm^3^). West Midlands curves are pooled from two measurement sites (Figure S2; https://links.lww.com/EE/A394).

Plots of the exposure-response relationship in secondary analyses allowing nonlinearity (Figure 1) also did not suggest evidence of an association between PNC and mortality. The shape of the curve was inconsistent between sites, with relatively wide confidence intervals (CIs) that include the null for all sites and outcomes, at all concentrations.

**Figure 2.**
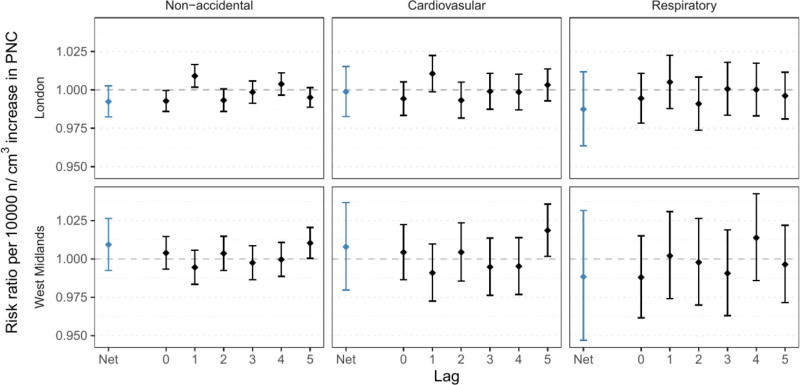
Secondary analysis on the short-term association between UFP and nonaccidental and cause-specific mortality. Cumulative and single-day (lagged 0–5 days) associations and 95% confidence intervals for a 10,000 particle/cm^3^ in PNC and nonaccidental and cause-specific mortality in London and the West Midlands. West Midlands results are pooled between two measurement sites.

Increasing the lagged period to 0–5 days did not notably alter estimated risk ratios (Figure 2, Table S2; https://links.lww.com/EE/A394). Some individual lag days could suggest an association, such as lag 1 for nonaccidental and cardiovascular mortality in London, but these are counterbalanced by negative risks at lag 0 and 2, and we could not exclude that this pattern can be attributed to random chance and multiple testing. Extending the lag period for temperature to 21 days produced similarly null results to the main analysis (Table S3; https://links.lww.com/EE/A394).

**Figure 3.**
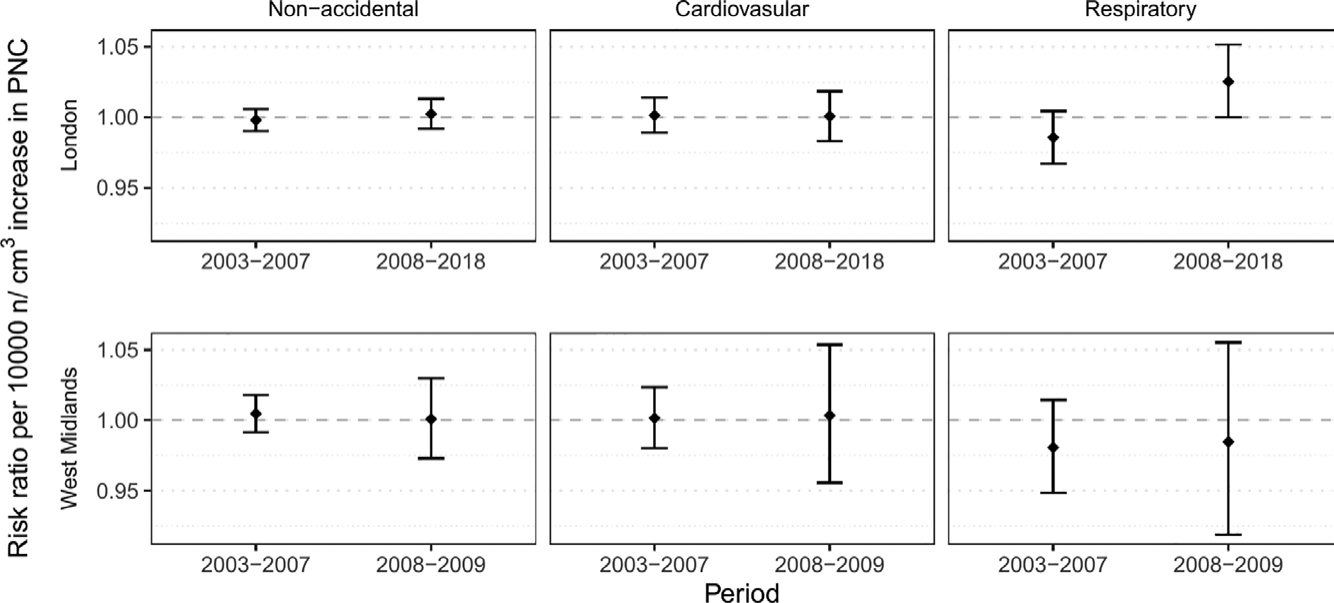
Secondary analysis on the effect modification of the 2007 “sulfur-free” fuel policy. Risk ratios of non-accidental and cause-specific mortality corresponding to a 10,000 n/cm^3^ increase in PNC, by location and time period (before or after the intervention), with a change point of 1 Jan 2008. West Midlands results are exclusively from the Birmingham Centre site.

The interrupted time-series analysis only suggested a change in risk for respiratory mortality in London (p_difference_ = 0.01) (Figure 3 and Table S4; https://links.lww.com/EE/A394). For a 10,000 particle/cm^3^ increase in PNC, the risk ratio was 0.986 (95% CI = 0.967, 1.005) before the change point and 1.025 (1.000, 1.052) after. We did not find evidence of a difference for any other outcome or location, and all risk ratios were similarly null.

The results for the bi-pollutant models with NO_2_ or PM_2.5_ are presented in Table S5 and Table S6; https://links.lww.com/EE/A394. Results remained null for all locations and outcomes, for both models.

The restricted analysis in the Royal Borough of Kensington and Chelsea produced null results for the main model and all secondary analyses (Table S7 and Figure S3; https://links.lww.com/EE/A394). It did not replicate the single-lag day associations seen in Greater London, and it showed no evidence of a change in risk after the 2007 fuel policy.

## Discussion

This study investigated the mortality risks associated with exposure to UFP in two large metropolitan areas in the UK. It found little evidence that suggests UFP affects mortality risk. We found limited evidence that the 2007 fuel policy modifies this association. We observed an increase in the risk after the change point for respiratory mortality in London only, and null results for all other outcomes and locations. It is possible this apparent increase is due to changes in the composition of the particles, as average UFP concentrations decreased after the change point. However, the evidence for effect modification is weak, considering the null main effect and the lack of replication in the West Midlands or in the geographically restricted analysis in Kensington.

The null results of this study align with a recently published systematic review and meta-analysis.^27^ The meta-analysis found summary estimates of 1.000 (95% CI = 0.993, 1.007), 0.996 (0.990, 1.002), and 1.005 (0.979, 1.032) for natural, cardiovascular, and respiratory mortality, respectively, per 10,000 particle/cm3 increase in UFP at lag 0, based on 8–10 studies per outcome. Summary estimates at lag 0–1, based on the results of 3–4 studies per outcome, were similarly null. This review, along with three previous reviews and the 2021 WHO Global Air Quality Guidelines, concludes that the current evidence base is inconclusive on the health effects of UFP.^2,6,8,27^ The reviews report that some past studies show positive associations, but the evidence base is limited by heterogeneity in model specification, exposure assessment methods, and outcome selection. Furthermore, the inconsistency in past results might be linked to geographic and composition differences that can alter the health impacts of UFP, as has been observed for PM_2.5_ and PM_10_.^28^ It is not uncommon for analyses in the UK, particularly, to produce inconclusive or null results for PM and mortality.^19^

Our results are somewhat consistent with the two previous studies in London.^9,10^ Both studies used data from the same North Kensington measurement site used in this analysis, though time periods and modeling specifications varied. Samoli and colleagues^9^ reported no evidence suggestive of an association between UFP and mortality for any tested mortality outcome between 2011–2012. For 2000–2005, Atkinson and colleagues^10^ reported increases in daily mortality from all causes (1.4%, 95% CI = 0.5%, 2.4%), cardiovascular causes (2.2%, 95% CI = 0.6%, 3.8%), and respiratory causes (2.3%, 95% CI = −0.1%, 4.8%) for a 10,166 particle/cm^3^ increase in PNC lagged 1 day, though with null results at all other lags and no a priori reason to suspect an effect at this specific lag. We find a similar pattern across lags in London, but this did not propagate to the overall cumulative effect. Such a pattern can be influenced by the inherent instability of unconstrained distributed lag models, which are not well adapted to accurately estimate lag-response functions.^29^

It is possible that other traffic policies affected the analysis of the effect modification of the 2007 fuel policy. The London Low Emission Zone was introduced in May 2007, 1 month before the enactment of the “sulfur-free” fuel policy.^11^ We focused on the fuel policy, as Jones and colleagues,^11^ who identified the reduction in UFP concentrations, concluded that the fuel policy was likely the main driver in the reduction in observed UFP concentrations since the reduction was also observed in Birmingham, which had no Low Emission Zone at the time.

This study has several strengths, most notably the long study period relative to previous publications on the topic and the multi-location design that both increase its power. In comparison to previous publications, which had an average study period of 7 years, we used a combined 25 years of data from two metropolitan areas, amounting to a total of 851,726 deaths.

A notable limitation of this study is the use of UFP data from a single monitoring site in each conurbation. This simple exposure assessment method could lead to exposure misclassification, which can dilute effect estimates. However, while the spatial variability of UFP means that this data would be inadequate to capture individual-level exposures across the conurbations, previous studies have demonstrated moderately-high to high temporal correlations of UFP measurements between sites.^30–32^ As concluded in Cyrys et al.,^30^ these high correlations suggest that centrally located monitoring sites are adequate for epidemiological time-series studies, as they capture the temporal contrasts needed for the study design. While we were unable to internally confirm this temporal correlation in this study due to data limitations, Harrison et al.^32^ reported substantial similarities in temporal patterns across five measurement sites in London, including the North Kensington site used in this analysis. As an additional validation step, we conducted a restricted analysis in a smaller area of London close to the central monitoring site, which confirmed our null results.

Further limitations of this study include the measurement site change in the West Midlands in 2009 and the considerable amount of missing data (>30% of days), which both constrain the statistical power of this analysis. Additionally, we were not able to incorporate data on UFP size into the model due to data limitations. UFP size can be an indicator of source, which could impact the composition and toxicity of the particles.^7^

## Conclusions

This study is the largest known analysis of the relationship between UFP air pollution and mortality in England, to date, drawing from a combined 25 years of data in two cities. It is also the first known study to investigate the possible effect-modifying role of the UK “sulfur-free” diesel fuel policy in 2007. We found no evidence that suggested that UFP was associated with nonaccidental, cardiovascular, or respiratory death in London and the West Midlands from 2003 to 2018, and little evidence that the fuel policy modified this association. However, these results are hampered by possibly important exposure misclassification due to the use of a single monitor per city. This reinforces the need for a larger monitoring network and more advanced exposure assessment methods.

## Conflicts of interest statement

The authors declare that they have no conflicts of interest with regard to the content of this report.

## Acknowledgements

The individual mortality data used to create the daily time series were provided by the UK ONS through data access agreement MRP 229/2013. The authors would like to thank David Butterfield at National Physical Laboratory (NPL) for advice on the past operation of PNC measurement equipment; the scientists at NPL and Imperial College London, who undertook the PNC measurements; and Defra, who funded and provided the PNC measurements.

## Supplementary Material
