# Supplementary material for: Mortality risks associated with short-term exposure to ultrafine particles in London and the West Midlands
Source: Environ Epidemiol. 2025 Dec 16;10(1):e449. doi: 10.1097/EE9.0000000000000449 (PMC12711358; doi:10.1097/EE9.0000000000000449)
Supplement: Supplementary file 1 [file ee9-10-e449-s001.pdf]

Supplementary materials

Methods

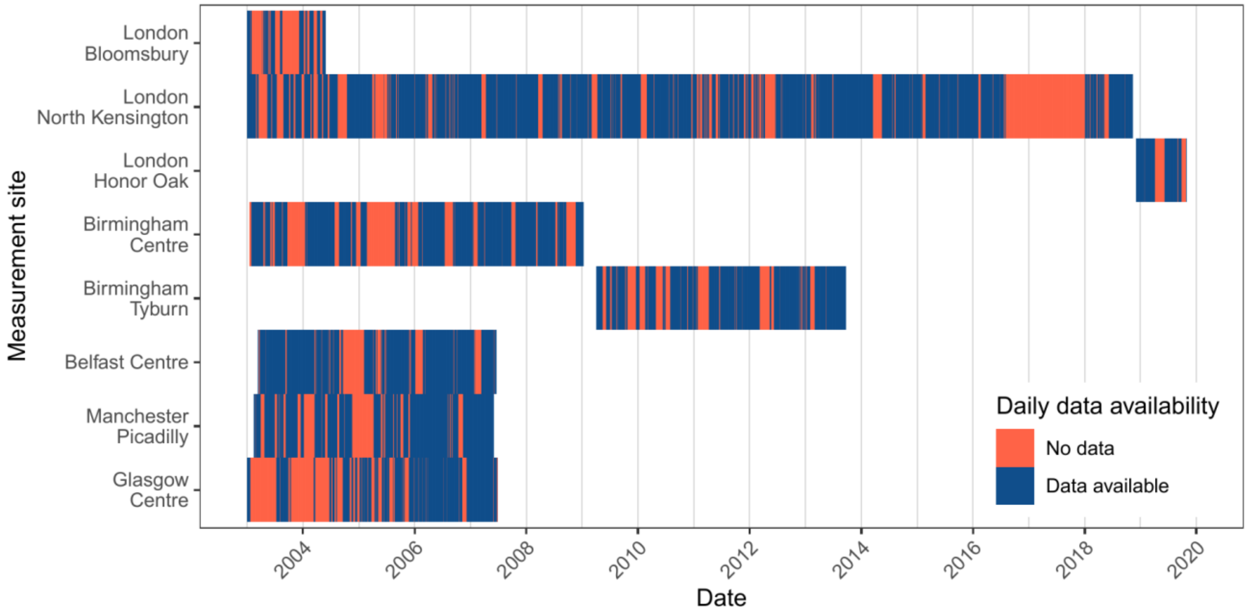

Figure S1: Length of UFP measurement and data missing patterns for all urban background measurement sites for UFP in the UK.

Model selection for secondary analysis of nonlinearity

We selected a natural cubic spline with a single knot placed at the median PNC across all sites (13160 particles/cm^3) after conducting a model selection process testing b-splines and natural splines with varying numbers and placements of knots. We selected the optimal model based on minimization of a modified Akaike's Information Criterion for over dispersed count data (QAIC). Boundary knots are placed at the minimum and maximum UFP values across all sites, excluding an outlier in the West Midlands.

Results

Table S1: Relative risk and 95% confidence interval for the West Midlands, by measurement site and pooled, associated with a 10,000 particle/cm^3 increase in PNC over 0-1 days with adjustment for seasonality, long-term trend, and temperature.

	Birmingham Centre	Birmingham Tyburn	Pooled
Non-accidental	1.003 (0.991 to 1.015)	0.994 (0.974 to 1.015)	1.001 (0.990 to 1.011)
Cardiovascular	0.998 (0.978 to 1.018)	0.973 (0.938 to 1.010)	0.992 (0.975 to 1.010)
Respiratory	0.985 (0.954 to 1.017)	1.010 (0.958 to 1.065)	0.991 (0.965 to 1.019)

Table S2: Cumulative and single-day (lagged 0-5 days) associations and 95% confidence intervals for a 10,000 particle/cm3 in PNC and non-accidental and cause-specific mortality in London and the West Midlands.

	Non-accidental			
	London	Birmingham Centre	Birmingham Tyburn	Pooled West Midlands
**net**	**0.992 (0.982,1.003)**	**1.015 (0.996,1.034)**	**0.987 (0.955,1.021)**	**1.008 (0.992,1.025)**
lag0	0.993 (0.986,1.000)	1.003 (0.993,1.013)	0.996 (0.976,1.017)	1.001 (0.992,1.011)
lag1	1.009 (1.002,1.016)	0.995 (0.982,1.007)	0.998 (0.976,1.019)	0.996 (0.985,1.006)
lag2	0.993 (0.986,1.001)	1.004 (0.991,1.017)	1.003 (0.981,1.025)	1.003 (0.992,1.015)
lag3	0.999 (0.991,1.006)	1.004 (0.991,1.017)	0.981 (0.960,1.002)	0.998 (0.987,1.009)
lag4	1.004 (0.997,1.011)	0.999 (0.987,1.012)	1.001 (0.980,1.022)	1.000 (0.989,1.011)
lag5	0.995 (0.989,1.002)	1.010 (0.999,1.022)	1.009 (0.989,1.028)	1.010 (1.000,1.020)
	Cardiovascular			
	London	Birmingham Centre	Birmingham Tyburn	Pooled West Midlands
**net**	**0.999 (0.983,1.015)**	**1.012 (0.980,1.045)**	**0.982 (0.926,1.040)**	**1.005 (0.977,1.033)**
lag0	0.994 (0.983,1.005)	1.001 (0.984,1.018)	0.991 (0.956,1.027)	0.998 (0.983,1.014)
lag1	1.011 (0.999,1.022)	0.997 (0.976,1.019)	0.982 (0.946,1.020)	0.993 (0.975,1.012)
lag2	0.993 (0.982,1.005)	1.000 (0.979,1.022)	1.015 (0.977,1.055)	1.004 (0.986,1.023)
lag3	0.999 (0.987,1.011)	1.003 (0.982,1.025)	0.970 (0.934,1.008)	0.995 (0.977,1.014)
lag4	0.999 (0.987,1.010)	0.998 (0.977,1.019)	0.991 (0.954,1.029)	0.996 (0.977,1.014)
lag5	1.003 (0.993,1.014)	1.013 (0.993,1.032)	1.033 (0.998,1.069)	1.018 (1.001,1.036)
	Respiratory			
	London	Birmingham Centre	Birmingham Tyburn	Pooled West Midlands
**net**	**0.987 (0.964,1.012)**	**0.990 (0.943,1.040)**	**0.991 (0.910,1.079)**	**0.992 (0.951,1.035)**
lag0	0.994 (0.978,1.011)	0.998 (0.971,1.025)	0.983 (0.933,1.036)	0.993 (0.970,1.018)
lag1	1.005 (0.988,1.023)	0.992 (0.960,1.025)	1.015 (0.961,1.072)	0.999 (0.972,1.028)
lag2	0.991 (0.974,1.008)	0.986 (0.954,1.019)	1.030 (0.976,1.088)	0.998 (0.970,1.027)
lag3	1.001 (0.983,1.018)	1.016 (0.984,1.050)	0.925 (0.875,0.978)	0.991 (0.963,1.019)
lag4	1.000 (0.983,1.017)	1.010 (0.977,1.043)	1.026 (0.972,1.083)	1.013 (0.985,1.042)
lag5	0.996 (0.981,1.012)	0.989 (0.960,1.018)	1.016 (0.967,1.067)	0.997 (0.972,1.022)

Table S3: Relative risk and 95% confidence interval associated with a 10,000 particle/cm^3 increase in PNC over 0-1 days with adjustment for seasonality, long-term trend, and temperature (lagged 21 days).

	London	West Midlands[a]
Non-accidental	1.000 (0.993 to 1.008)	1.005 (0.994 to 1.016)
Cardiovascular	1.006 (0.994 to 1.018)	0.997 (0.979 to 1.016)
Respiratory	0.990 (0.972 to 1.008)	0.989 (0.962 to 1.017)

[a]pooled

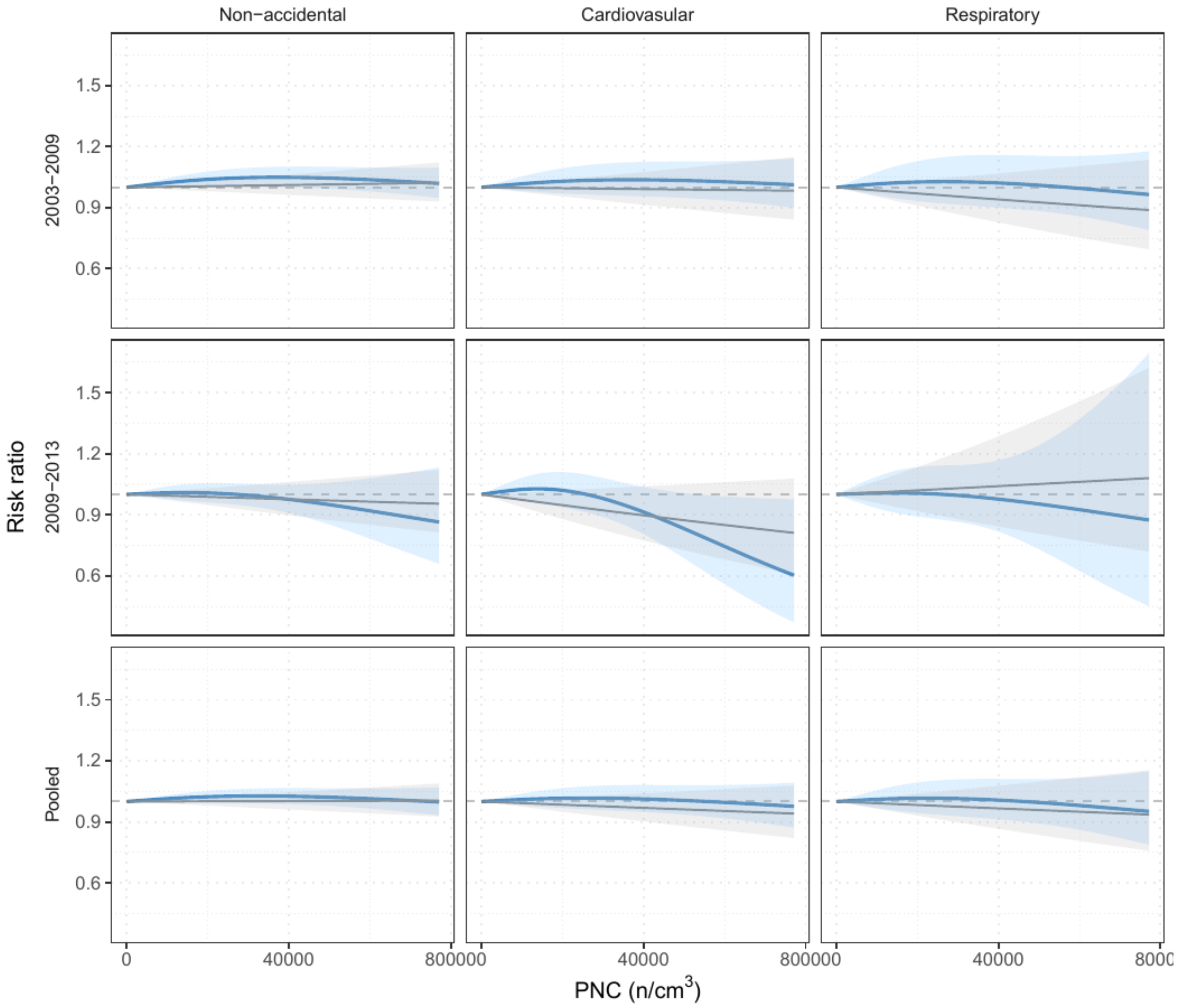

Figure S2: Exposure-response curves for the West Midlands, by measurement site and pooled. Estimates obtained using a linear term (grey line) or a natural cubic spline (blue line) with a single knot placed at the median PNC (13,160 n/cm^3).

Table S4: Secondary analysis on the effect modification of the 2007 "sulfur-free" fuel policy. Risk ratios of non-accidental and cause specific mortality corresponding to a 10,000 n/cm^3 increase in PNC, by location and time period (before or after the intervention), with a change point of 1 Jan, 2008. West Midlands results are from the Birmingham Centre site.

		Prior to change point	After change point	p-value[a]
London	Non-accidental	0.998 (0.990, 1.006)	1.003 (0.992, 1.013)	0.47
	Cardiovascular	1.001 (0.989, 1.014)	1.001 (0.983, 1.019)	0.95
	Respiratory	0.986 (0.967, 1.005)	1.025 (1.000, 1.052)	0.01
West Midlands	Non-accidental	1.005 (0.992, 1.018)	1.001 (0.973, 1.030)	0.79
	Cardiovascular	1.002 (0.980, 1.023)	1.003 (0.956, 1.054)	0.94
	Respiratory	0.981 (0.948, 1.014)	0.985 (0.919, 1.055)	0.91

[a]from X^2 test of a difference

Table S5: Relative risk and 95% confidence interval from bi-pollutant model (UFP, NO_2) associated with a 10,000 particle/cm^3 increase in PNC over 0-1 days with adjustment for seasonality, long-term trend, and temperature.

Mortality cause	Pollutant	Location	
		London	West Midlands[a]
Non-accidental	UFP	0.993 (0.983 to 1.003)	1.005 (0.993 to 1.017)
	NO_2	1.004 (1.000 to 1.009)	0.994 (0.986 to 1.003)
Cardiovascular	UFP	1.005 (0.989 to 1.021)	1.002 (0.981 to 1.022)
	NO_2	0.998 (0.990 to 1.005)	0.987 (0.973 to 1.001)
Respiratory	UFP	0.990 (0.967 to 1.015)	0.995 (0.964 to 1.027)
	NO_2	1.005 (0.994 to 1.016)	0.995 (0.974 to 1.016)

[a]pooled

Table S6: Relative risk and 95% confidence interval from bi-pollutant model (UFP, $PM_{2.5}$) associated with a 10,000 particle/cm^3 increase in PNC over 0-1 days with adjustment for seasonality, long-term trend, and temperature.

Mortality cause	Pollutant	Location	
		London	West Midlands[a]
Non-accidental	UFP	0.998 (0.990 to 1.005)	1.004 (0.993 to 1.015)
	$PM_{2.5}$	1.003 (0.997 to 1.009)	0.988 (0.976 to 1.000)
Cardiovascular	UFP	1.003 (0.991 to 1.016)	0.996 (0.978 to 1.014)
	$PM_{2.5}$	0.996 (0.986 to 1.006)	0.984 (0.963 to 1.006)
Respiratory	UFP	0.991 (0.973 to 1.009)	0.992 (0.964 to 1.020)
	$PM_{2.5}$	1.014 (1.000 to 1.028)	0.999 (0.968 to 1.031)

[a]pooled

Table S7: Relative risk and 95% confidence interval for the Greater London and the Royal Borough of Kensington and Chelsea associated with a 10,000 particle/cm^3 increase in PNC over 0-1 days with adjustment for seasonality, long-term trend, and temperature.

	London (main analysis)	Kensington and Chelsea
Non-accidental	0.998 (0.992 to 1.005)	1.005 (0.959 to 1.053)
Cardiovascular	1.000 (0.989 to 1.011)	0.945 (0.866 to 1.03)
Respiratory	0.997 (0.980 to 1.013)	0.962 (0.833 to 1.11)

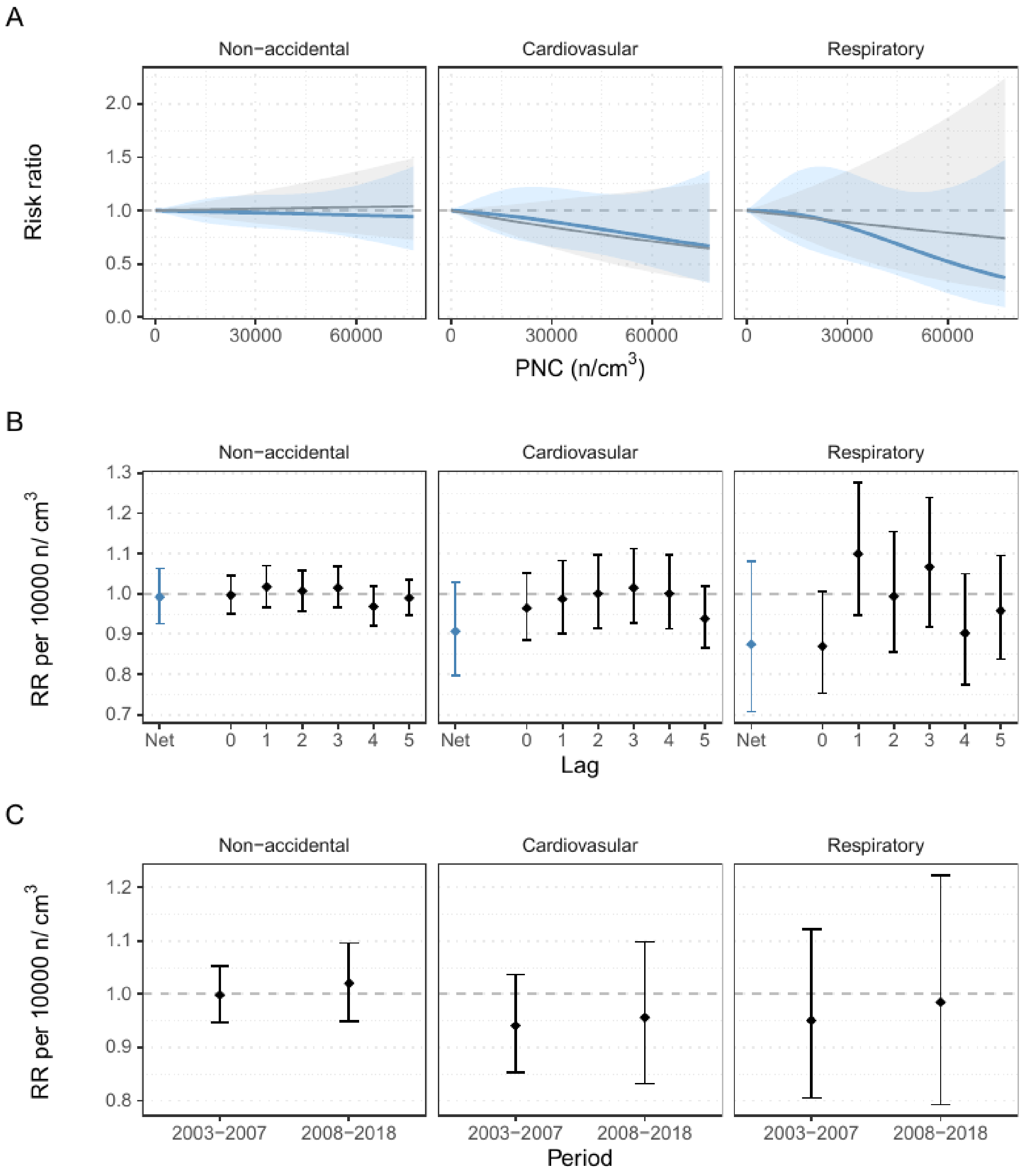

Figure S3: Associations between UFP and non-accidental and cause specific mortality in the Royal Borough of Kensington and Chelsea. A: Exposure response curves and 95% CI for linear (grey) and natural spline (blue) models. B: Cumulative and single-day lag associations and 95% CI for extended lag analysis. C: Associations before and after 2007 “sulfur-free” diesel fuel policy.
